# Frequent pain in older people with and without diabetes – Finnish community based study

**DOI:** 10.1186/s12877-018-0762-y

**Published:** 2018-03-15

**Authors:** M. Karjalainen, J. Saltevo, M. Tiihonen, M. Haanpää, H. Kautiainen, P. Mäntyselkä

**Affiliations:** 1 Institute of Public Health and Clinical Nutrition, General Practice, University of Eastern Finland, Kuopio, Finland; 2 Inner Savo Health Center, Suonenjoki, Finland; 3 Central Finland Central Hospital, Jyväskylä, Finland; 4 School of Pharmacy, University of Eastern Finland, P.O. BOX 1627, FI-70211 Kuopio, Finland; 5 Etera Mutual Pension Insurance Company, Vantaa, Finland; 6 Department of Neurosurgery, Helsinki University Hospital, Helsinki, Finland; 7 Unit of Primary Health Care, Helsinki University Central Hospital, Helsinki, Finland; 8 Primary Health Care Unit, Kuopio University Hospital, Kuopio, Finland

**Keywords:** Diabetes, Older people, Pain

## Abstract

**Background:**

The association between pain and diabetes in older people has been largely unexplored. The aim of this survey was to analyze the prevalence and characteristics of pain among Finnish men and women 65 or older with and without diabetes in primary care.

**Methods:**

All home-dwelling persons 65 years or older with diabetes (*N* = 527) and age and gender matched controls (*N* = 890) were identified from electronic patient records. Frequent pain was regarded as any pain experienced more often than once a week, and it was divided into pain experienced several times a week but not daily and pain experienced daily or continuously. The Numeric Rating Scale (0–10) (NRS) was used to assess the intensity and interference of the pain.

**Results:**

The number of subjects who returned the questionnaire was 1084 (76.5%). The prevalence of frequent pain in the preceding week was 50% among women without diabetes and 63% among women with diabetes (adjusted, *p* = 0.22). In men, the corresponding proportions were 42% without diabetes and 47% with diabetes (adjusted, *p* = 0.58). In both genders, depressive symptoms and the number of comorbidities were associated with pain experienced more often than once a week and with daily pain. Diabetes was not associated with pain intensity or pain interference in either women or men.

**Conclusions:**

Pain in older adults is associated with depressive symptoms and the number of comorbidities more than with diabetes itself.

## Background

Diabetes is among the most common chronic diseases in the world and in Finland [1]. The global prevalence of diabetes has nearly doubled from 1980 to 2014, increasing from 5% to 9% in the adult population [2]. In Finland the prevalence of diabetes is estimated to be 11%. Most patients with diabetes in Finland have type 2 diabetes (89%) [3]. The increasing incidence and prevalence of diabetes will inevitably result in accumulation of diabetes in older people [4]. It is assumed that people with diabetes have a bigger load of diseases than people without diabetes [5].

One important comorbid condition often linked to diabetes is chronic pain. In general, it is very common in the adult population [6, 7]. Chronic pain may be related to general multimorbidity [8] and even to mortality [9]. Patients with type 2 diabetes have an increased risk of developing specific rheumatic manifestations caused by diabetes, such as stiff hand syndrome, Dupuytren’s disease, tenosynovitis, carpal tunnel syndrome, shoulder capsulitis/periarthritis, and reduced joint mobility. In addition to the conditions probably caused by diabetes, obesity and physical inactivity may predispose to osteoarthritis [10], which therefore most likely is associated with rather than caused by the disease.

A clinically important complication of diabetes is neuropathy, which can be painful. The prevalence of neuropathic pain in people with diabetes is difficult to estimate, as definitions have varied enormously between studies. In an observational study of a large cohort of patients with diabetes in the U.K. the prevalence of painful neuropathy symptoms was estimated to be as high as 34% [11]. Furthermore, non-neuropathic pain is common among patients with diabetes [12].

Data on the pain of older people with diabetes are few. Cross-sectional data from a multi-site, prospective cohort study of 11,689 participants with diabetes aged 47–73 years in the United States found that moderate to extreme pain was present in 58% and pain was strongly associated with poorer mental health and physical functioning but not poorer glycemic control [13]. Another population study based on in-person interviews of adults 65 years old or older in the USA found that bothersome pain in the last month was reported by half of the adult population, while the corresponding prevalence among people with diabetes was 61.5% [14]. In Taiwan, a large population-based, retrospective cohort study found that people aged 18–50 years with type 2 diabetes had a higher 10-year cumulative incidence of and a higher mean number of doctor visits for musculoskeletal pain than a non-diabetic group [15]. Regardless of the etiology, musculoskeletal complaints are frequent among patients with diabetes mellitus type 2 and may be of major importance in terms of quality of life [16].

Both diabetes and chronic pain are more common among older people than younger people. It can be assumed that older people with diabetes may suffer pain and be affected by pain more than those without diabetes. However, the association between diabetes and pain in old people with a control group of people without diabetes has not been studied much. Therefore, the aim of this study was to analyze the prevalence, frequency, intensity and interfering effect of pain among women and men aged 65 or more with and without diabetes in a community-based population setting.

## Methods

### Study population

This cross-sectional study is based on ISDM (Inner-Savo Diabetes Mellitus) data. The data is obtained from the Inner-Savo district with a total population of 10,793. The present study was designed to collect data from a semirural community in order to have information for planning the services for older inhabitants. The study was approved by the Inner Savo Health Care Federation of Municipalities (61 A/2015). The study protocol of the ISDM study was approved by the Research Ethics Committee of the Northern Savo Hospital District, Kuopio, Finland (256/2015). The questionnaire included information letter about the use of data and returning of questionnaire was voluntary. The autonomy of research subjects was respected and only anonymous data were analyzed. No harm was possible for the subjects and confidentiality of the subjects and research data were protected. Of the inhabitants, 3093 (29%) were 65 or older representing a semi-rural area of Finland with a larger proportion of older people than in larger cities and average in Finland (20%) [17].

**Fig. 1:**
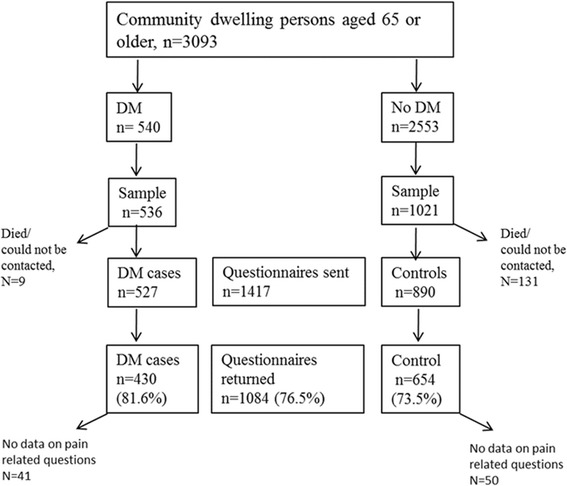
Flow chart of the study

Home-dwelling, 65 years or older persons with diabetes (and with Haemoglobin A1c (HbA1c) –levels) were identified from primary care electronic patient records using the International Classification of Diseases (ICD-10) diagnostic codes E10 and E11 [18]. Because only 12 subjects had type 1 diabetes people with type 1 and type 2 diabetes were combined. For each person with a diagnosis of diabetes, two control persons matched by age and gender were identified (Fig. 1). A total of 1417 questionnaires were handed out to 527 persons with diabetes and 890 persons without diabetes from August to September 2015. The study participants filled out a structured questionnaire including background variables, e.g., gender, age, and living arrangements (living alone or with someone, in a rural or urban area, in their own house, an apartment, or in supported living).

### Measurements and tools

Pain was defined as any pain experienced in the preceding week [7, 19]. Localizations of pain were recorded according to a list (head, thorax, abdomen, neck, shoulder, arm, other region of the upper limb, low back, hip, knee, other region of the lower limb, somewhere else). Pain experienced more often than once a week was regarded as frequent pain. Further, frequent pain was divided into pain experienced several times a week but not daily and pain experienced daily or continuously. Thus, three categories of pain were used in the analyses; (1) no frequent pain; (2) pain several times a week; and (3) daily or continuous pain. The Numeric Rating Scale (NRS) was used to assess the intensity and general interference of the pain with the general activity [20]. Respondents were asked to rate their pain intensity and interference on a scale 0 to 10, with 0 being no pain or no interference at all and 10 being the worst imaginable pain or a complete interference. The duration of pain was asked and chronic pain was defined as pain with the duration of at least 3 months.

The questionnaire included lifestyle items regarding physical activity based on the Kasari-FIT index with three questions on the frequency, efficiency, and duration of exercise [21]. We measured lifestyle items regarding alcohol consumption and smoking with the Alcohol Use Disorders Identification Test (AUDIT-C) [22]. An alcohol user was defined as a person using alcohol at least once a month. To assess depressive symptoms, the 15-item Geriatric Depression Scale (GDS-15) was used [23].

The subjects were asked to report long-term diseases they had according to a list including the most common chronic diseases: High blood pressure, cardiovascular disease, cancer, rheumatoid arthritis, osteoarthritis, chronic spine disease, asthma/COPD, mental illness, hypothyroidism. The reported diseases were summed up to represent the number of chronic diseases (number of comorbidities). In the subjects with diabetes, the number of comorbidities means those in addition to diabetes. The mean HbA1c level was based on the recordings of the electronic patient record data in the preceding year.

#### Statistical analysis

Because men and women are potentially different in terms of pain and diabetes, all the analyses were conducted separately for men and women. The baseline sample characteristics are presented as amounts and percentages for categorical variables and means with standard deviations for continuous variables. Statistical comparisons between the groups were made using the t-test, chi-square test, or analysis of variance. Multinominal (polytomous) logistic regression analyses were performed to identify demographic, clinical, and functional factors associated with weekly and daily pain. A bootstrap method was used when the theoretical distribution of the test statistics was unknown or in the case of a violation of the assumptions (e.g. non-normality). Adjusted curvilinear relationships between HbA1c and intensity and interference of pain were derived from regression models including quadratic term of HbA1C. Bootstrap estimation was used to derive a 95% confidence interval of adjusted curvilinear correlation. The normality of the variables was tested by using the Shapiro-Wilk W test. No adjustment for multiple comparisons was considered necessary. The Stata 14.1, StataCorp LP (College Station, TX, USA) statistical package was used for the analysis.

## Results

A total of 1084 (76.5%) questionnaires were returned (Fig. 1). Among the subjects with diabetes, the response rate was 81.6% (*N* = 430) and among the controls, respectively, 73.5% (*N* = 654) (*p* < 0.001). Complete data were available for 993 participants (70.0% of the sample).

The characteristics of the study subjects are shown in Table 1. Females with diabetes were 2 years older than the controls. A slightly but significantly smaller proportion of men with diabetes lived in a detached house compared with men without diabetes. Ability to move without aid and physical activity were lower and hypertension and cardiovascular disease and number of comorbidities were higher among persons with diabetes in both genders. Smoking was less common among men with diabetes than among controls, while women used less alcohol than those without diabetes. Compared with people without diabetes, women with diabetes had more depressive symptoms.

**Table 1:** Characteristics of the participating women and men with and without diabetes

	Women			Men		
	No diabetes *N* = 329	Diabetes *N* = 198	*P*-value	No diabetes *N* = 275	Diabetes *N* = 191	*P*-value
Age, mean (SD)	75 (7)	77 (8)	0.002	73 (5)	74 (6)	0.26
Living in a detached house, n (%)	256 (78)	144 (73)	0.19	239 (87)	152 (80)	0.034
Living in the countryside, n (%)	90 (28)	54 (29)	0.85	109 (40)	70 (38)	0.60
Living alone, n (%)	132 (40)	95 (48)	0.078	60 (22)	44 (23)	0.76
Physical activity, Kasari-FIT Index, mean (SD)	38 (22)	26 (19)	< 0.001	43 (23)	33 (23)	< 0.001
Smoking, n (%)	16 (5)	6 (3)	0.31	45 (16)	15 (8)	0.007
Alcohol use, AUDIT-C, mean (SD)	1.34 (1.45)	0.92 (1.33)	< 0.001	2.92 (2.58)	2.75 (2.35)	0.49
Depressive symptoms, GDS-15, mean (SD)	2.5 (2.8)	3.8 (3.0)	< 0.001	2.6 (3.0)	3.1 (3.0)	0.13
Comorbidities, n (%)						
High blood pressure	180 (55)	149 (75)	< 0.001	128 (47)	129 (68)	< 0.001
Cardiovascular disease	44 (13)	47 (24)	0.002	45 (16)	40 (21)	0.21
Cancer	20 (6)	10 (5)	0.62	20 (7)	13 (7)	0.85
Rheumatoid arthritis	19 (6)	9 (5)	0.54	10 (4)	6 (3)	0.77
Osteoarthritis	97 (29)	73 (37)	0.079	62 (23)	50 (26)	0.37
Chronic spine disease	95 (29)	55 (28)	0.79	52 (19)	44 (23)	0.28
Asthma/COPD	45 (14)	34 (17)	0.28	20 (7)	22 (12)	0.12
Mental illness	40 (12)	32 (16)	0.20	22 (8)	19 (10)	0.46
Hypothyroidism	71 (22)	53 (27)	0.17	17 (6)	15 (8)	0.48
Number of comorbidities, mean (SD)	1.9 (1.4)	2.3 (1.5)	< 0.001	1.4 (1.3)	1.8 (1.4)	0.002
Any pain, n (%)	236 (72)	163 (82)	0.006	193 (70)	129 (68)	0.054
Pain localization, n (%)						
Low back	120 (36)	83 (42)	0.21	68 (25)	52 (27)	0.54
Shoulder	71 (22)	74 (34)	< 0.001	66 (24)	49 (26)	0.68
Knee	98 (30)	77 (39)	0.032	60 (22)	43 (23)	0.86
Hip	81 (25)	64 (32)	0.055	47 (17)	37 (19)	0.53
Other lower extremity	77 (23)	61 (31)	0.061	44 (16)	36 (19)	0.42
Neck	101 (31)	54 (27)	0.40	59 (21)	42 (22)	0.89
Other upper extremity	22 (7)	24 (12)	0.032	15 (5)	10 (5)	0.92

*SD* Standard deviation, *Kasari-FIT index* person’s level of physical activity (Frequency, Intensity, Time), *Alcohol user* alcohol use at least once a month according to the Alcohol Use Disorders Identification Test (AUDIT-C), *GDS-15* Geriatric Depression Scale, *COPD* chronic obstructive pulmonary disease

In general, women with diabetes experienced pain more often than women without diabetes. A corresponding difference was not found in men. Women with diabetes more often had pain in their shoulders, knees, and upper extremities than women without diabetes. There were no corresponding significant differences in pain localizations in men.

**Fig. 2:**
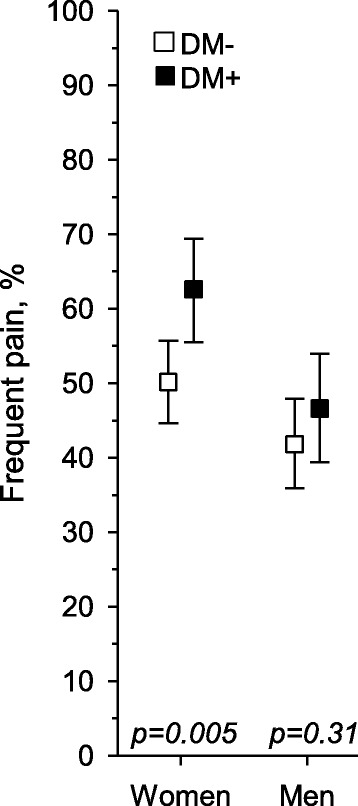
Prevalence of frequent pain among women and men with and without diabetes (*p*-values are crude, the corresponding *p* values adjusted for age, physical activity, depressive symptoms, alcohol use, smoking and number of comorbidities were *p* = 0.22 in women and *p* = 0.58 in men)

Frequent pain was experienced less often by women without diabetes than by those with diabetes (Fig. 2). However, after adjustment for age, physical activity, depressive symptoms, alcohol use, smoking, and number of comorbidities, no difference was found. In men, there was not any significant difference in the presence of frequent pain. Among the women with frequent pain, chronic pain was found in 94% (155/165) of those without diabetes and in 94% (116/124) with diabetes (*p* = 0.89). Respectively, in men, pain was chronic in 91% without diabetes and 94% with diabetes (*p* = 0.40).

**Fig. 3:**
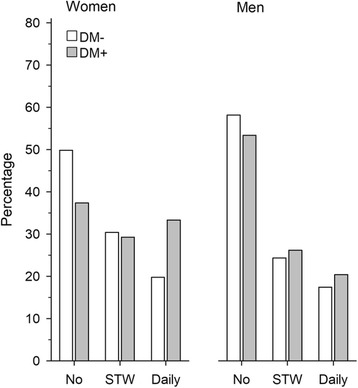
Proportions of subjects without frequent pain, with pain several times a week (STW), and with daily pain among women and men with and without diabetes

The distribution of pain according to frequency is presented in Fig. 3. The crude prevalence of daily pain in women was 19.8% (*N* = 65) without diabetes and 33.3% (*N* = 66) with diabetes (*p* = 0.002). The corresponding numbers in men were in 17.5% (*N* = 48) without diabetes and 20.4% (*N* = 39) with diabetes (*p* = 0.34).

Table 2 shows the results of multinominal regression analysis conducted separately for women and men assessing the association of several variables with frequent pain. The model included diabetes (no diabetes as a reference), age (per 1-SD), depressive symptoms (GDS-15 score per 1-SD), physical activity (Kasari FIT Index per 1-SD), alcohol use (AUDIT-C per 1-SD), smoking (no smoking as reference) and number of comorbidities. In both women and men, depressive symptoms and the number of comorbidities were associated with pain experienced several times a day and daily pain. Diabetes was not associated with pain in either women or men. Neither age, depressive symptoms, physical activity, alcohol use nor smoking were associated with frequent pain.

**Table 2:** Factors associated with frequent pain. Results of the multinomial logistic regression analysis. Reference group: no frequent pain

	Pain more often than once a week RRR^a^ (95% CI:)	*P*-value	Pain daily or continuously RRR^a^ (95% CI:)	*P*-value
Women				
Diabetes	1.09 (0.67 to 1.75)	0.73	1.44 (0.84 to 2.47)	0.18
Age / 1-SD	0.90 (0.70 to 1.15)	0.39	0.98 (0.74 to 1.30)	0.89
GDS-15 / 1-SD	1.49 (1.11 to 1.98)	0.007	1.96 (1.44 to 2.65)	< 0.001
Kasari-FIT Index / 1-SD	0.98 (0.76 to 1.26)	0.85	0.84 (0.61 to 1.14)	0.26
AUDIT-C / 1-SD	1.18 (0.94 to 1.48)	0.16	1.24 (0.95 to 1.63)	0.12
Smoking	1.04 (0.35 to 3.04)	0.95	0.94 (0.26 to 3.41)	0.93
Number of comorbidities	1.52 (1.28 to 1.82)	< 0.001	1.93 (1.59 to 2.35)	< 0.001
Men				
Diabetes	0.92 (0.56 to 1.52)	0.74	1.15 (0.64 to 2.07)	0.64
Age / 1-SD	1.04 (0.80 to 1.34)	0.78	1.12 (0.82 to 1.51)	0.48
GDS-15 / 1-SD	1.34 (1.00 to 1.79)	0.047	2.12 (1.56 to 2.87)	< 0.001
Kasari-FIT Index / 1-SD	0.85 (0.65 to 1.12)	0.25	1.27 (0.92 to 1.74)	0.14
AUDIT-C / 1-SD	1.16 (0.90 to 1.48)	0.24	1.04 (0.77 to 1.41)	0.80
Smoking	1.19 (0.60 to 2.38)	0.62	0.49 (0.18 to 1.37)	0.17
Number of comorbidities	1.49 (1.22 to 1.82)	< 0.001	1.84 (1.47 to 2.30)	< 0.001

*SD* Standard deviation, *GDS-15* Geriatric Depression Scale, *Kasari-FIT index* person’s level of physical activity (Frequency, Intensity, Time), *AUDIT-C* Alcohol Use Disorders Identification Test

^a^Relative Risk Ratio

**Fig. 4:**
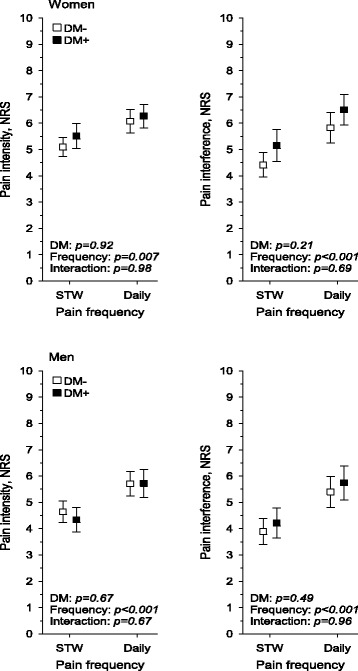
Intensity and interference of pain experienced several times a week (STW) and pain experienced daily among women and men without and with diabetes

**Fig. 5:**
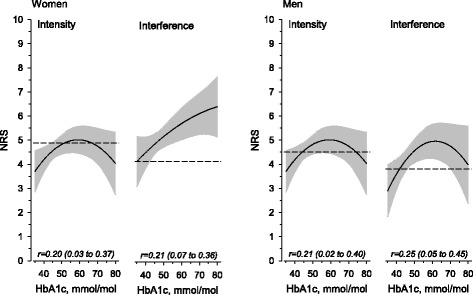
Relationships between HbA1c and intensity and interference of pain among women and men with diabetes. The curves were derived from regression models including quadratic term of HbA1c. The curves were adjusted for age, physical activity, depressive symptoms, alcohol use, smoking and number of comorbidities. The grey area represents 95% confidence intervals. Dotted lines show means of subjects without diabetes. Adjusted curvilinear correlation was used. All curvilinear correlations were significant

Figure 4 presents the pain intensity and interference assessed with NRS. Diabetes was not associated with pain interference or intensity in either women or men. Pain intensity and interference were experienced higher in daily pain compared with less frequent pain in women and men. There was not any interaction in intensity or interference of pain between diabetes and pain frequency in women and men. Among persons with diabetes, significant association was found between pain intensity, interference, and HbA1C (Fig. 5).

## Discussion

This primary care setting study found a high prevalence of frequent pain in older people with and without diabetes. In both women and men, there were no significant differences in the adjusted prevalence of frequent pain between those with and without diabetes. Diabetes did not explain the presence of frequent pain in either women or men, but comorbidities and depressive symptoms did. Neither was diabetes associated with the intensity or interference of pain. Furthermore, in women, there were differences in the reported localizations of pain between those without and with diabetes.

The prevalence of pain found in the present study is quite similar to that found in previous studies of older people [15, 19, 24]. For example, a recent population-based study from Sweden with people aged 60 years and older reported the prevalence pain as 55% [25]. Respectively, the prevalence of daily chronic pain was almost 30% in people aged 65–74 years in another population-based study [7]. In general, the prevalence of chronic pain has been found to be 50% or more among people aged 65 years or older [24]. In people with diabetes, women have more neuropathic pain than men [11]. A difference in pain occurrence and characteristics between subjects with and without diabetes has also been reported [15, 24]. In the present study, osteoarthritis was more common in women with diabetes, although there was no statistical difference. Knee osteoarthritis [26] and upper extremity pain [27] are more common in people with diabetes. In a meta-analysis of 18 selected articles, it was found that people with diabetes were five times more likely to have adhesive capsulitis in their shoulders than controls [28]. Therefore, the higher prevalence of knee and shoulder pain and the higher crude prevalence of daily pain in women with diabetes compared with women without diabetes could be partly explained by the higher rate of knee osteoarthritis and chronic shoulder disorders associated with diabetes.

In the present study we were not able to detect or diagnose neuropathic pain. Based on previous studies exploring the occurrence of neuropathic pain in diabetic people, it could have been assumed that frequent pain would have been clearly more prevalent among diabetic people also in the present study. However, we could not detect any significant difference between the subjects without or with diabetes. Contrary to previous assumptions, this makes one hypothesize that neuropathic pain in older people is not associated with diabetes. As previously stated, we were not able to analyze neuropathic pain in the present study. Therefore, we are not able to exclude the possibility that, compared with women without diabetes, the more prevalent daily pain in those with diabetes may be due to neuropathy. However, we can conclude that, in general, frequent pain is not associated with diabetes in older home-dwelling people. The HbA1c level was associated with the intensity and interference of pain. The subjects in the present study represent home-dwelling people with probably at least moderately controlled disease. Therefore, it is possible that among patients with a worse treatment situation the prevalence of pain could be higher.

The present study indicates that comorbid diseases and depressive symptoms are more significant than diabetes in pain. People with diabetes more often have depressive symptoms and depression [13, 29]. On the other hand, depression and chronic pain often co-occur [13]. Furthermore, the burden of diseases among people with diabetes is heavier [4, 30, 31].

The strength of our study is the population-based study sample that comprehensively represents older people in a primary care setting with diabetes based on a recorded diagnosis. The response rate was high.

Our study can be considered epidemiological rather than clinical and it has also limitations. The study population was from one primary care district and therefore the generalization of these results is only possible in older Finnish semi-rural population. Our study had a cross-sectional design, therefore a cause-effect relationship cannot be presumed. Pain and other characteristics were based on self-reported data, which may be prone to inaccuracy. Although, our sample was based on the confirmed diagnosis of diabetes, the presence of other chronic diagnoses was based on questionnaire data. We were not able to define the onset or the progression of diabetes precisely although we were able to obtain the mean HbA1c values from the patient record. The subjects were collected according to the established diagnosis of diabetes 3 months before the questionnaire was sent. Therefore, the study sample did not include any recently diagnosed patients and we can assume that these subjects represent home-dwelling primary care patients with a substantially long history of diabetes. On the other hand, the participants of the present study do not represent frailer older people or persons with further progressed diabetes who do not live at home.

## Conclusions

The findings of the present study showed that more than with diabetes itself, pain in older adults is associated with depressive symptoms and the burden of diseases. This implies that health care professionals have to consider throughout assessment and personal health care plans for home-dwelling older people suffering from frequent pain with and without diabetes.

## Abbreviations

AUDIT-C: Alcohol Use Disorders Identification Test
COPD: Chronic Obstructive Pulmonary Disease
GDS-15: Geriatric Depression Scale
HbA1c: Haemoglobin A1c
ICD-10: International Classification of Diseases
ISDM: Inner-Savo Diabetes Mellitus
NRS: The Numeric Rating Scale
SD: Standard deviation
